# *N*′-Cyclo­pentyl­idene­pyridine-4-carbo­hydrazide

**DOI:** 10.1107/S1600536812013815

**Published:** 2012-04-04

**Authors:** Andreas Lemmerer, Joel Bernstein, Volker Kahlenberg

**Affiliations:** a Molecular Sciences Institute, School of Chemistry, University of the Witwatersrand, Johannesburg, PO Wits 2050, South Africa; b Faculty of Science, NYU Abu Dhabi, PO Box 129188, Abu Dhabi, United Arab Emirates; c Institute of Mineralogy and Petrography, University of Innsbruck, Innsbruck 6020, Austria

## Abstract

The title compound, C_11_H_13_N_3_O, is a derivative of the anti­tuberculosis drug isoniazid [systematic name: pyridine-4-carbohydrazide]. The crystal structure consists of repeating hydrogen-bonded chains parallel to the *b* axis. Adjacent mol­ecules in the chains are linked by bifurcated N—H⋯(O,N) hydrogen bonds, which form an *R*_1_^2^(5) ring motif.

## Related literature

For hydrogen-bond motifs, see: Bernstein *et al.* (1995 ▶).
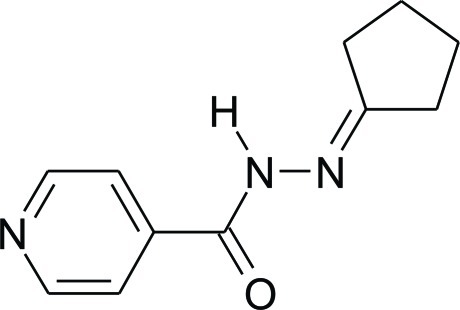


## Experimental

### 

#### Crystal data

C_11_H_13_N_3_O
*M* *_r_* = 203.24
Orthorhombic, 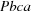

*a* = 15.762 (3) Å
*b* = 8.1144 (16) Å
*c* = 16.015 (3) Å
*V* = 2048.3 (7) Å^3^
*Z* = 8
Mo *K*α radiation
μ = 0.09 mm^−1^
*T* = 173 K
0.35 × 0.22 × 0.2 mm

#### Data collection

Oxford Diffraction Xcalibur Gemini R diffractometer
Absorption correction: multi-scan (ABSPACK in *CrysAlis PRO*; Oxford Diffraction, 2006 ▶) *T*_min_ = 0.970, *T*_max_ = 0.983
11794 measured reflections
1884 independent reflections
1420 reflections with *I* > 2σ(*I*)
*R*_int_ = 0.034

#### Refinement

*R*[*F* ^2^ > 2σ(*F* ^2^)] = 0.032
*wR*(*F* ^2^) = 0.081
*S* = 0.96
1884 reflections
140 parameters
H atoms treated by a mixture of independent and constrained refinement
Δρ_max_ = 0.14 e Å^−3^
Δρ_min_ = −0.21 e Å^−3^

### 

Data collection: *CrysAlis PRO* (Oxford Diffraction, 2006 ▶); cell refinement: *CrysAlis PRO*; data reduction: *CrysAlis PRO*; program(s) used to solve structure: *SHELXS97* (Sheldrick, 2008 ▶); program(s) used to refine structure: *SHELXL97* (Sheldrick, 2008 ▶); molecular graphics: *ORTEP-3 for Windows* (Farrugia, 1997 ▶) and *DIAMOND* (Brandenburg, 1999 ▶); software used to prepare material for publication: *WinGX* (Farrugia, 1999 ▶) and *PLATON* (Spek, 2009 ▶).

## Supplementary Material

Crystal structure: contains datablock(s) global, I. DOI: 10.1107/S1600536812013815/fy2050sup1.cif

Supplementary material file. DOI: 10.1107/S1600536812013815/fy2050Isup2.mol

Structure factors: contains datablock(s) I. DOI: 10.1107/S1600536812013815/fy2050Isup3.hkl

Supplementary material file. DOI: 10.1107/S1600536812013815/fy2050Isup4.cml

Additional supplementary materials:  crystallographic information; 3D view; checkCIF report

## Figures and Tables

**Table 1:** Hydrogen-bond geometry (Å, °)

*D*—H⋯*A*	*D*—H	H⋯*A*	*D*⋯*A*	*D*—H⋯*A*
N1—H1⋯N3^i^	0.889 (15)	2.303 (15)	3.1155 (16)	151.9 (12)
N1—H1⋯O1^i^	0.889 (15)	2.607 (15)	3.3368 (14)	140.0 (11)

Symmetry code: (i) 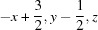.

**Fig. 1.**
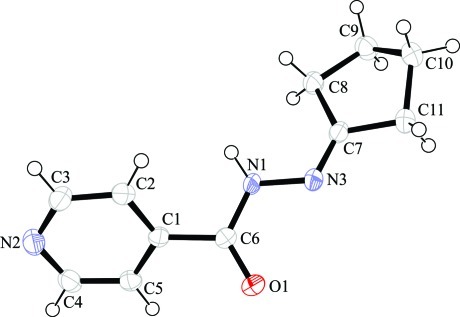
The asymmetric unit of (I) showing the atom numbering scheme. Displacement ellipsoids are shown at the 50% probability level.

**Fig. 2.**
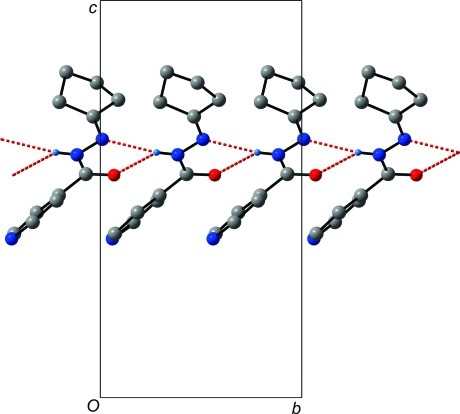
Hydrogen bonding chain showing the ring shaped hydrogen bonding motif. Intermolecular N—H···O and N—-H···N hydrogen bonds are shown as dashed red lines.

## supplementary crystallographic information

### Comment

Fig. 1 shows the atomic numbering scheme of the title compound. The amide functional groups form a torsion angle of -39.73 (17)° (C5—C1—C6—O1) with the pyridine ring. Fig. 2 shows the *R*^2^_1_(5) (Bernstein *et al.*, 1995) hydrogen bonded ring formed with adjacent amide functional groups, leading to a chain along the *b*-axis.

### Experimental

A stoichiometric amount in the ratio of 1:1 of isonicotinic acid hydrazide (0.200 g, 1.46 mmol) to cyclopentanone (0.129 g) was dissolved in 5 ml of methanol. The solution was refluxed for a few hours, and left to cool to room temperature. Colourless, block-like crystals were harvested after slow evaporation over a few days at ambient conditions.

### Refinement

The C-bound H atoms were geometrically placed (C—H bond lengths of 0.95 (aromatic CH) and 0.99 (methylene CH_2_) Å) and refined as riding with *U*_iso_(H) = 1.2*U*_eq_(C). The N-bound H atoms were located in the difference map and coordinates refined freely together with their isotropic thermal parameters.

### Crystal data

**Table:**

C_11_H_13_N_3_O	*F*(000) = 864
*M**_r_* = 203.24	*D*_x_ = 1.318 Mg m^−^^3^
Orthorhombic, *P**b**c**a*	Mo *K*α radiation, λ = 0.71073 Å
Hall symbol: -P 2ac 2ab	Cell parameters from 5050 reflections
*a* = 15.762 (3) Å	θ = 2.9–28.5°
*b* = 8.1144 (16) Å	µ = 0.09 mm^−^^1^
*c* = 16.015 (3) Å	*T* = 173 K
*V* = 2048.3 (7) Å^3^	Block, colourless
*Z* = 8	0.35 × 0.22 × 0.2 mm

### Data collection

**Table:**

Oxford Diffraction Xcalibur Gemini R diffractometer	1420 reflections with *I* > 2σ(*I*)
ω scans	*R*_int_ = 0.034
Absorption correction: multi-scan (ABSPACK in *CrysAlis PRO*; Oxford Diffraction, 2006)	θ_max_ = 25.5°, θ_min_ = 3.1°
*T*_min_ = 0.970, *T*_max_ = 0.983	*h* = −17→18
11794 measured reflections	*k* = −9→9
1884 independent reflections	*l* = −19→14

### Refinement

**Table:**

Refinement on *F*^2^	0 restraints
Least-squares matrix: full	H atoms treated by a mixture of independent and constrained refinement
*R*[*F*^2^ > 2σ(*F*^2^)] = 0.032	*w* = 1/[σ^2^(*F*_o_^2^) + (0.0527*P*)^2^] where *P* = (*F*_o_^2^ + 2*F*_c_^2^)/3
*wR*(*F*^2^) = 0.081	(Δ/σ)_max_ < 0.001
*S* = 0.96	Δρ_max_ = 0.14 e Å^−^^3^
1884 reflections	Δρ_min_ = −0.21 e Å^−^^3^
140 parameters	

### Special details

**Table:**

Geometry. All e.s.d.'s (except the e.s.d. in the dihedral angle between two l.s. planes) are estimated using the full covariance matrix. The cell e.s.d.'s are taken into account individually in the estimation of e.s.d.'s in distances, angles and torsion angles; correlations between e.s.d.'s in cell parameters are only used when they are defined by crystal symmetry. An approximate (isotropic) treatment of cell e.s.d.'s is used for estimating e.s.d.'s involving l.s. planes.

### Fractional atomic coordinates and isotropic or equivalent isotropic displacement parameters (Å^2^)

**Table:**

	*x*	*y*	*z*	*U*_iso_*/*U*_eq_	
C1	0.63373 (8)	0.29380 (15)	0.50919 (7)	0.0229 (3)	
C2	0.68605 (8)	0.18488 (16)	0.46629 (8)	0.0300 (3)	
H2	0.7458	0.1879	0.4736	0.036*	
C3	0.64926 (9)	0.07249 (17)	0.41303 (9)	0.0362 (3)	
H3	0.6856	−0.0007	0.3836	0.043*	
C4	0.51676 (9)	0.16426 (16)	0.44216 (8)	0.0330 (3)	
H4	0.4571	0.1569	0.4345	0.04*	
C5	0.54713 (8)	0.28276 (16)	0.49627 (7)	0.0265 (3)	
H5	0.5093	0.3554	0.5241	0.032*	
C6	0.66894 (7)	0.42811 (15)	0.56313 (7)	0.0223 (3)	
C7	0.83244 (7)	0.46470 (15)	0.70795 (8)	0.0238 (3)	
C8	0.84781 (8)	0.29668 (16)	0.74453 (8)	0.0284 (3)	
H8A	0.8763	0.2236	0.7037	0.034*	
H8B	0.7939	0.245	0.7624	0.034*	
C9	0.90543 (8)	0.33071 (17)	0.81972 (9)	0.0322 (3)	
H9A	0.8715	0.3536	0.8705	0.039*	
H9B	0.9435	0.2361	0.8307	0.039*	
C10	0.95583 (8)	0.48203 (16)	0.79324 (8)	0.0302 (3)	
H10A	1.0022	0.4519	0.7544	0.036*	
H10B	0.9803	0.5393	0.8422	0.036*	
C11	0.88949 (8)	0.58868 (17)	0.74995 (9)	0.0328 (3)	
H11A	0.8576	0.6558	0.7909	0.039*	
H11B	0.9161	0.6629	0.7085	0.039*	
N1	0.73514 (6)	0.38419 (14)	0.61158 (6)	0.0247 (3)	
H1	0.7496 (8)	0.2789 (19)	0.6174 (9)	0.036 (4)*	
N2	0.56579 (8)	0.05969 (14)	0.40008 (7)	0.0377 (3)	
N3	0.78134 (6)	0.50934 (12)	0.65045 (6)	0.0251 (3)	
O1	0.63990 (5)	0.56802 (10)	0.56036 (5)	0.0298 (2)	

### Atomic displacement parameters (Å^2^)

**Table:**

	*U*^11^	*U*^22^	*U*^33^	*U*^12^	*U*^13^	*U*^23^
C1	0.0297 (7)	0.0192 (6)	0.0197 (6)	0.0008 (5)	−0.0020 (5)	0.0038 (5)
C2	0.0304 (7)	0.0305 (7)	0.0292 (7)	0.0059 (6)	−0.0038 (5)	−0.0017 (6)
C3	0.0478 (9)	0.0292 (8)	0.0316 (7)	0.0092 (7)	−0.0051 (6)	−0.0063 (6)
C4	0.0349 (7)	0.0283 (7)	0.0359 (8)	−0.0032 (6)	−0.0117 (6)	0.0051 (6)
C5	0.0295 (7)	0.0238 (7)	0.0260 (7)	0.0008 (6)	−0.0024 (5)	0.0041 (5)
C6	0.0232 (6)	0.0207 (7)	0.0230 (6)	0.0000 (5)	0.0028 (5)	0.0013 (5)
C7	0.0260 (7)	0.0200 (7)	0.0253 (7)	0.0003 (5)	0.0004 (5)	−0.0029 (5)
C8	0.0322 (7)	0.0218 (7)	0.0312 (7)	−0.0027 (6)	−0.0053 (5)	0.0016 (6)
C9	0.0335 (8)	0.0330 (8)	0.0299 (7)	−0.0018 (6)	−0.0052 (5)	0.0016 (6)
C10	0.0296 (7)	0.0332 (8)	0.0279 (7)	−0.0029 (6)	−0.0047 (5)	−0.0027 (6)
C11	0.0349 (8)	0.0244 (7)	0.0390 (8)	−0.0033 (6)	−0.0091 (6)	−0.0044 (6)
N1	0.0319 (6)	0.0146 (6)	0.0277 (6)	−0.0010 (5)	−0.0073 (4)	−0.0016 (5)
N2	0.0499 (8)	0.0289 (7)	0.0343 (6)	−0.0009 (6)	−0.0141 (5)	−0.0026 (5)
N3	0.0284 (6)	0.0189 (6)	0.0281 (6)	−0.0021 (4)	−0.0041 (4)	−0.0023 (5)
O1	0.0298 (5)	0.0221 (5)	0.0374 (5)	0.0048 (4)	−0.0036 (4)	−0.0022 (4)

### Geometric parameters (Å, º)

**Table:**

C1—C5	1.3836 (18)	C7—C11	1.5076 (17)
C1—C2	1.3904 (18)	C8—C9	1.5333 (18)
C1—C6	1.4973 (17)	C8—H8A	0.99
C2—C3	1.3768 (19)	C8—H8B	0.99
C2—H2	0.95	C9—C10	1.5227 (19)
C3—N2	1.3360 (19)	C9—H9A	0.99
C3—H3	0.95	C9—H9B	0.99
C4—N2	1.3309 (18)	C10—C11	1.5243 (18)
C4—C5	1.3801 (18)	C10—H10A	0.99
C4—H4	0.95	C10—H10B	0.99
C5—H5	0.95	C11—H11A	0.99
C6—O1	1.2249 (14)	C11—H11B	0.99
C6—N1	1.3484 (16)	N1—N3	1.3961 (14)
C7—N3	1.2759 (15)	N1—H1	0.889 (15)
C7—C8	1.5035 (17)		
			
C5—C1—C2	118.04 (11)	C9—C8—H8B	111
C5—C1—C6	119.94 (11)	H8A—C8—H8B	109
C2—C1—C6	121.87 (11)	C10—C9—C8	103.63 (10)
C3—C2—C1	118.51 (12)	C10—C9—H9A	111
C3—C2—H2	120.7	C8—C9—H9A	111
C1—C2—H2	120.7	C10—C9—H9B	111
N2—C3—C2	124.23 (13)	C8—C9—H9B	111
N2—C3—H3	117.9	H9A—C9—H9B	109
C2—C3—H3	117.9	C9—C10—C11	103.09 (10)
N2—C4—C5	124.11 (13)	C9—C10—H10A	111.1
N2—C4—H4	117.9	C11—C10—H10A	111.1
C5—C4—H4	117.9	C9—C10—H10B	111.1
C4—C5—C1	118.76 (12)	C11—C10—H10B	111.1
C4—C5—H5	120.6	H10A—C10—H10B	109.1
C1—C5—H5	120.6	C7—C11—C10	103.49 (10)
O1—C6—N1	123.71 (11)	C7—C11—H11A	111.1
O1—C6—C1	121.01 (11)	C10—C11—H11A	111.1
N1—C6—C1	115.24 (10)	C7—C11—H11B	111.1
N3—C7—C8	129.82 (11)	C10—C11—H11B	111.1
N3—C7—C11	120.59 (11)	H11A—C11—H11B	109
C8—C7—C11	109.59 (10)	C6—N1—N3	117.90 (10)
C7—C8—C9	103.78 (10)	C6—N1—H1	120.9 (9)
C7—C8—H8A	111	N3—N1—H1	121.2 (9)
C9—C8—H8A	111	C4—N2—C3	116.34 (11)
C7—C8—H8B	111	C7—N3—N1	116.39 (10)
			
C5—C1—C2—C3	0.30 (18)	C7—C8—C9—C10	−30.69 (13)
C6—C1—C2—C3	−175.20 (12)	C8—C9—C10—C11	40.97 (13)
C1—C2—C3—N2	−0.6 (2)	N3—C7—C11—C10	−163.83 (11)
N2—C4—C5—C1	−0.92 (19)	C8—C7—C11—C10	15.97 (14)
C2—C1—C5—C4	0.40 (17)	C9—C10—C11—C7	−34.78 (13)
C6—C1—C5—C4	175.99 (11)	O1—C6—N1—N3	−10.45 (17)
C5—C1—C6—O1	−39.73 (17)	C1—C6—N1—N3	167.52 (9)
C2—C1—C6—O1	135.69 (13)	C5—C4—N2—C3	0.65 (19)
C5—C1—C6—N1	142.25 (11)	C2—C3—N2—C4	0.1 (2)
C2—C1—C6—N1	−42.34 (16)	C8—C7—N3—N1	−3.79 (18)
N3—C7—C8—C9	−171.14 (12)	C11—C7—N3—N1	175.96 (10)
C11—C7—C8—C9	9.09 (14)	C6—N1—N3—C7	166.86 (11)

### Hydrogen-bond geometry (Å, º)

**Table:**

*D*—H···*A*	*D*—H	H···*A*	*D*···*A*	*D*—H···*A*
N1—H1···N3^i^	0.889 (15)	2.303 (15)	3.1155 (16)	151.9 (12)
N1—H1···O1^i^	0.889 (15)	2.607 (15)	3.3368 (14)	140.0 (11)

Symmetry code: (i) −*x*+3/2, *y*−1/2, *z*.
